# Supplementary material for: Neuronal parts list and wiring diagram for a visual system
Source: Nature. 2024 Oct 2;634(8032):166–80. doi: 10.1038/s41586-024-07981-1 (PMC11446827; doi:10.1038/s41586-024-07981-1)
Supplement: Supplementary file 5 — Cards for all cell types. Each card has 6 panels (clockwise): logical connectivity predicate diagram, numeric stats, illustration of a typical cell, followed by 3D rendering of all cells from three orthogonal viewpoints. Also available at Codex. [file 41586_2024_7981_MOESM5_ESM.zip › pdf/LPi10.pdf]

*LPi*10

predicate precision: 43%, recall: 100%

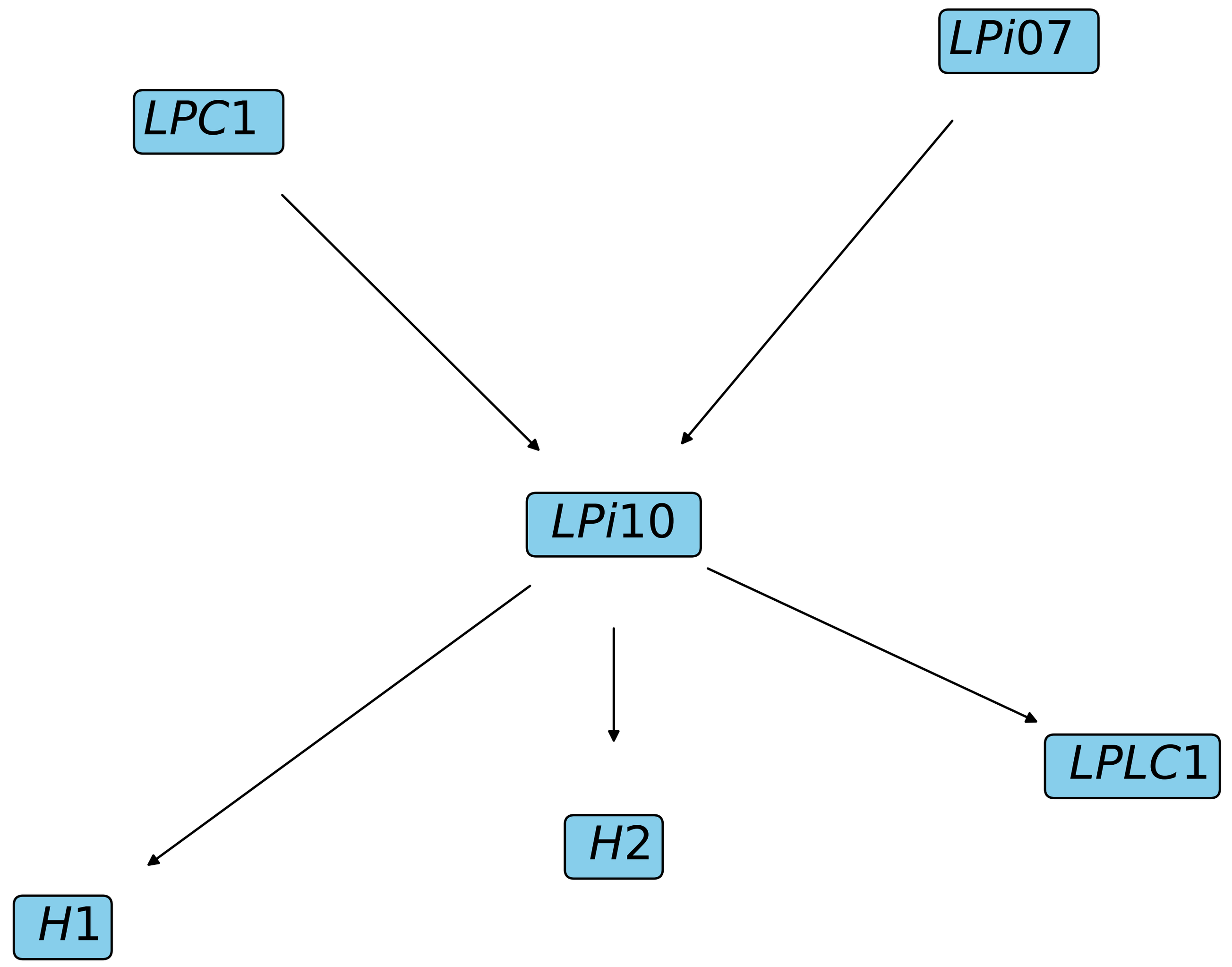

avg. stats

cells	21
NT prediction	GLUT (95%)
top in/out regions	LOP (100%) / LOP (100%)
in/out degree	336 / 309
in/out synapses	1,721 / 1,353
cable len	$3,061\mu$
surface	$4,522\mu^2$
volume	$235\mu^3$

morphology

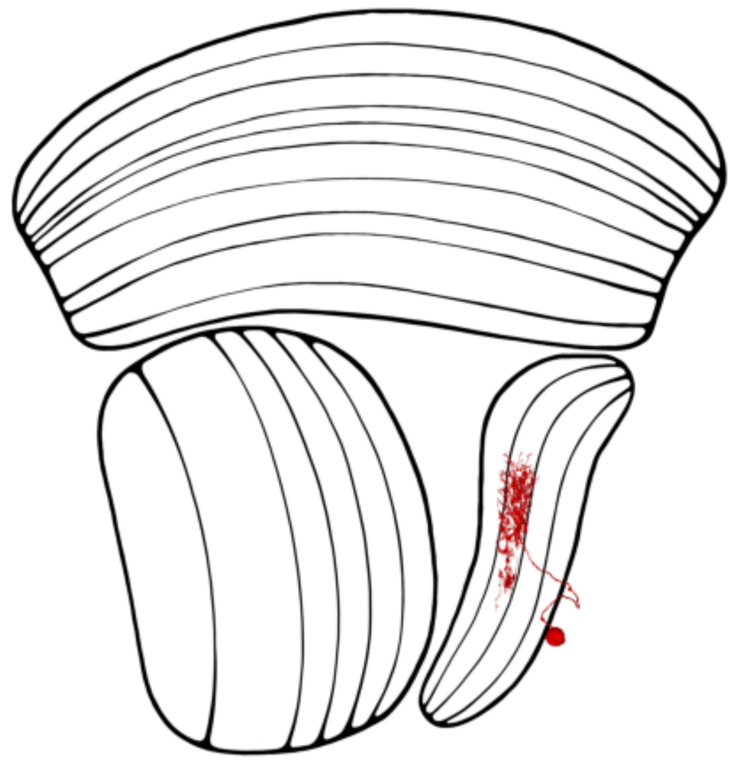

anterior view

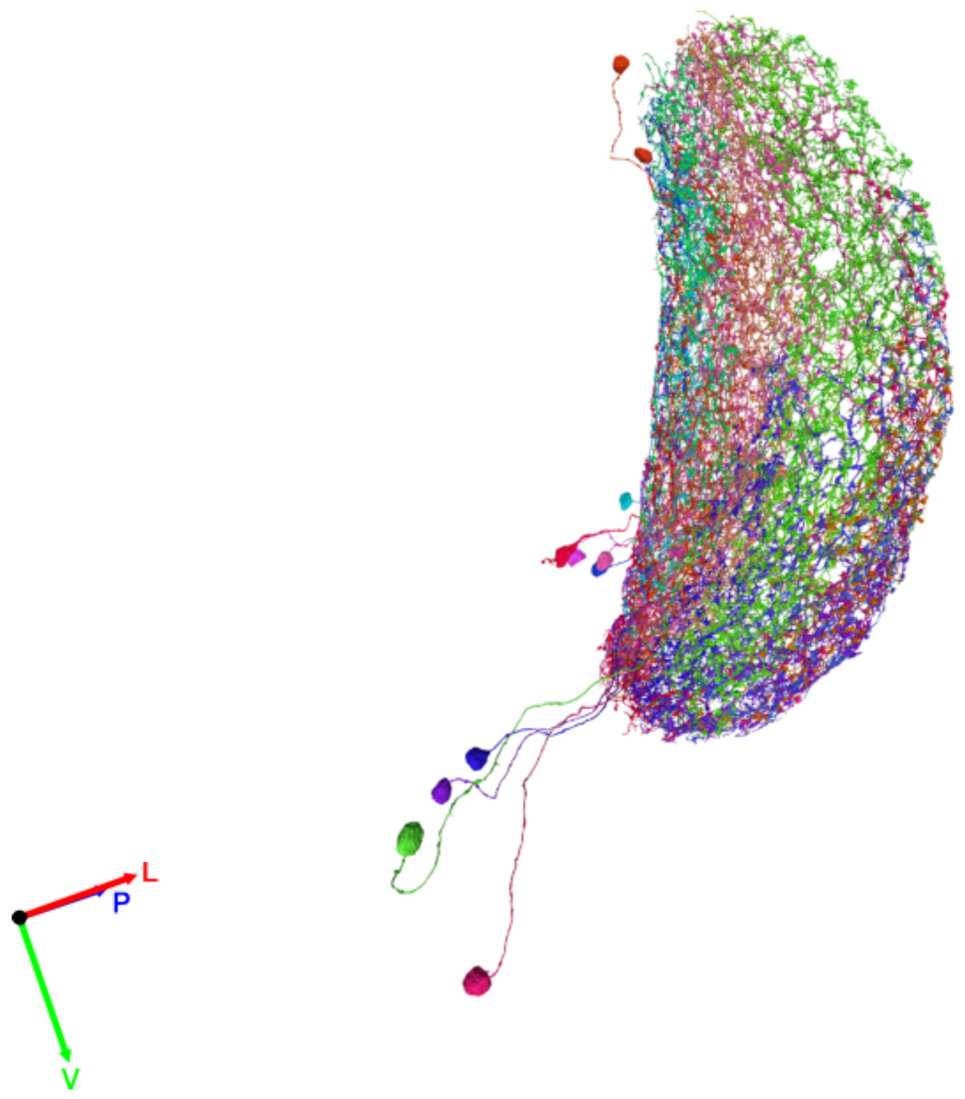

ventral view

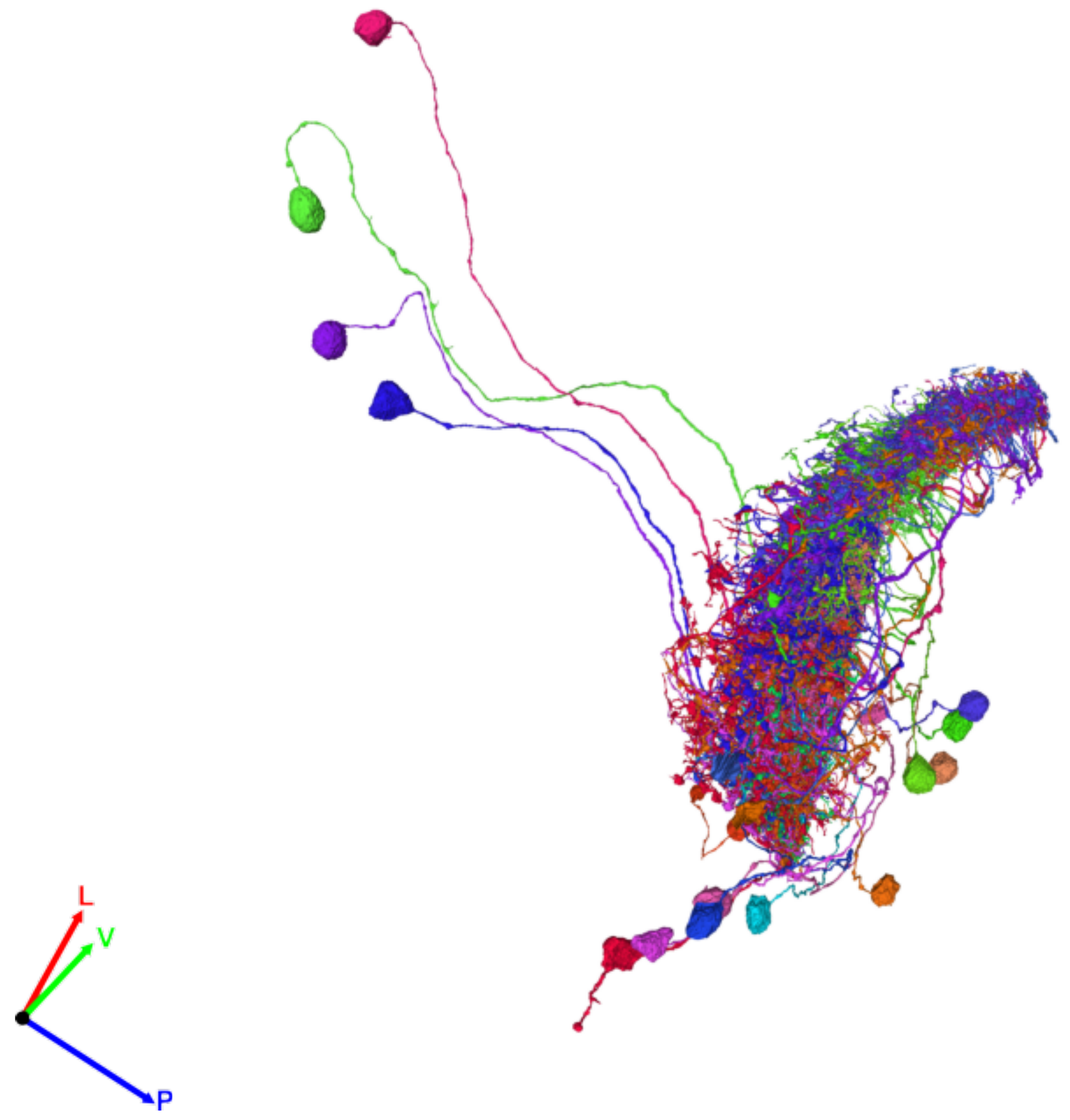

side view

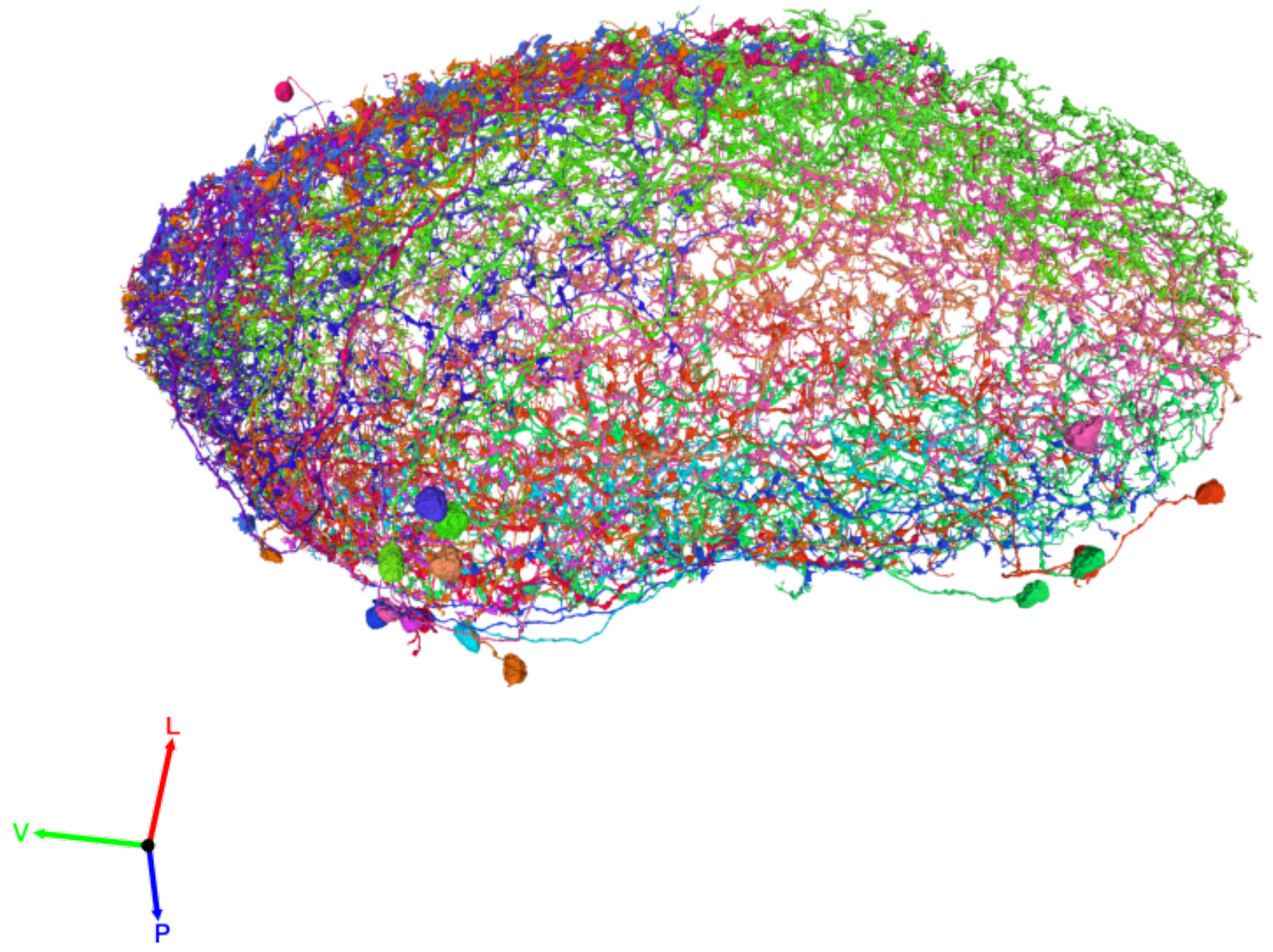

Lobula Plate Intrinsic
